# Developing a multimodal maternal infant perinatal outpatient delivery system: the MOMI PODS program

**DOI:** 10.3389/fgwh.2023.1232662

**Published:** 2023-09-21

**Authors:** Seuli Bose Brill, Lisa A. Juckett, Rachel D’Amico Gordon, Nikki Thomas, Alicia Bunger, Naleef Fareed, Christiane Voisin, Paola Flores, Shengyi Mao, Kristen L. Benninger, William Grobman, Bethany Panchal, Shannon Gillespie, Allison Lorenz

**Affiliations:** ^1^ Division of General Internal Medicine, Department of Internal Medicine, The Ohio State University College of Medicine, Columbus, OH, United States; ^2^ Division of Occupational Therapy, School of Health and Rehabitiation Sciences, The Ohio State University College of Medicine, Columbus, OH, United States; ^3^ Center for the Advancement of Team Science, Analytics, and Systems Thinking, The Ohio State University, Columbus, OH, United States; ^4^ College of Social Work, The Ohio State University, Columbus, OH, United States; ^5^ Department of Biomedical Informatics, The Ohio State University, Columbus, OH, United States; ^6^ Department of Neonatology, Nationwide Children’s Hospital, Columbus, OH, United States; ^7^ Division of Maternal Fetal Medicine, Department of Obstetrics and Gynecology, The Ohio State University College of Medicine, Columbus, OH, United States; ^8^ Department of Family and Community Medicine, The Ohio State University College of Medicine, Columbus, OH, United States; ^9^ College of Nursing, The Ohio State University, Columbus, OH, United States; ^10^ Ohio Colleges of Medicine Government Resource Center, The Ohio State University, Columbus, OH, United States

**Keywords:** maternal health, primary care, transition of care, implementation, preventive care, infrastructure, postpartum

## Abstract

Progress in maternal child health has been hampered by poor rates of outpatient follow up for postpartum individuals. Primary care after delivery can effectively detect and treat several pregnancy-related complications and comorbidities, but postpartum linkage to primary care remains low. In this manuscript, we share the experience of implementing a novel mother-infant dyad program, the Multimodal Maternal Infant Perinatal Outpatient Delivery System (MOMI PODS), to improve primary care linkage and community resource access postpartum via integration into pediatric care structures. With a focus on providing care for people who are publicly insured, we designed a program to mitigate maternal morbidity risk factors in postpartum individuals with chronic disease or pregnancy complications. We discuss the systematic process of designing, executing, and evaluating a collaborative clinical program with involvement of internal medicine/pediatric, family medicine, and obstetric clinicians via establishing stakeholders, identifying best practices, drawing from the evidence base, designing training and promotional materials, training partners and providers, and evaluating clinic enrollment. We share the challenges encountered such as in achieving sufficient provider capacity, consistent provision of care, scheduling, and data tracking, as well as mitigation strategies to overcome these barriers. Overall, MOMI PODS is an innovative approach that integrates outpatient postpartum care into traditional pediatric structures to increase access, showing significant promise to improve healthcare utilization and promote postpartum health.

## 1. Introduction

Fewer than half of postpartum individuals attend recommended clinical visits after childbirth (1). Inconsistent handoffs between obstetric clinicians and primary care providers (PCPs) create missed opportunities to improve health outcomes among postpartum individuals as well as their infants (2). These missed opportunities are particularly concerning for postpartum individuals with chronic disease or pregnancy-related health complications. Almost half of individuals with complicated pregnancies do not attend primary care visits in the 12 months after pregnancy (3, 4), which is a crucial opportunity for chronic disease prevention and screening. Innovative, patient-centered care models that transition individuals to primary care services after high-risk pregnancy have demonstrated the potential to address this gap in care (5). Concomitant parent and infant care can reduce coordination needed for parental appointments and further pre-established therapeutic relationships with providers. This dyadic primary care approach, in which mother and infant receive care within a unified medical home, also brings attention to pregnancy-related risk factors through the delivery of primary care services that meet the specific needs of postpartum individuals and infants, contextualized around their unique obstetric history (6).

To improve postpartum transitions from obstetric to primary care for individuals after high-risk pregnancy, we developed a health system-wide mother-infant dyadic care program [i.e., the Multimodal Maternal Infant Perinatal Outpatient Delivery System (MOMI PODS) program]. MOMI PODS represents a collaborative clinic structure involving clinicians from internal medicine/pediatrics, family medicine, and obstetrics, building upon a previous, disease-specific [i.e., gestational diabetes (GDM)] pilot program. Referrals from the community as well as obstetric providers were accepted for those whose history was notable for chronic or gestational diabetes, hypertension, depression, or who had a premature birth. In this paper, we (a) describe our systematic process of developing and implementing the MOMI PODS program and (b) present plans for program improvement after 12 months of implementation.

## 2. Context and key programmatic elements

The MOMI PODS program was created using a systematic intervention development process at a large academic medical center. Prior to MOMI PODS, obstetric clinicians provided postpartum follow up and while many family medicine and internal medicine/pediatric providers had the ability to care for both parents and children, there was no existing ability to schedule parents and infants together or integration of postpartum care and community resources. MOMI PODS design was informed by the Consolidated Framework for Implementation Research (CFIR) (7); CFIR guides comprehensive evaluation of multilevel implementation contexts and is well suited for rapid evaluation of complex healthcare delivery interventions such as MOMI PODS. CFIR contextualizes intervention characteristics, inner setting, outer setting, characteristics of target individuals, and the implementation process to promote a pragmatic approach to healthcare delivery interventions.

**Figure 1:**
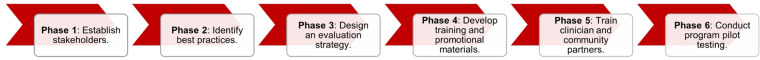
Phases of MOMI PODS implementation.

MOMI PODS design and implementation was characterized by six distinct phases. First, we established a MOMI PODS stakeholder group and identified current evidence and best practices for increasing access to postpartum outpatient care after high-risk pregnancy (Phases 1–3). This development process included seeking and incorporating patient feedback on the existing GDM dyad clinic, including the development of promotional and training materials (Phase 4), into the new MOMI PODS clinic model (6). Upon completion of provider training, we pilot tested MOMI PODS to determine the extent to which it improved access to postpartum primary care for the mother-baby dyad (Phases 5–6). Each of these six phases are described in detail below and depicted in Figure 1.

### 2.1. Phase 1: establish MOMI PODS stakeholders

Our initial group of MOMI PODS stakeholders consisted of four primary care physicians, two maternal-fetal medicine physicians, two obstetric physicians, three clinical nurse coordinators, two maternal-child health (MCH) policy experts, and three maternal-child health researchers. We also recruited patients known to the study team to serve on our stakeholder team to better understand barriers to accessing primary care in the postpartum setting and provide feedback (6). We incorporated feedback obtained from patients to help us form better partnerships, address gaps in patient care, and tailor to targeted individuals as guided by CFIR (7) through this co-design process. We included primary care providers from family medicine and internal medicine-pediatrics to provide comprehensive expertise on postpartum and infant care.

A total of 16 stakeholders held biweekly meetings between 06/2019 and 07/2021 to design the structure, operational resources, and distribution of shared resources for MOMI PODS, prior to its initial deployment. Following deployment, stakeholders continued to meet monthly. Separate data meetings were held to ensure adequate capture of clinical variables to support quality improvement, as well as monthly clinical meetings to discuss updates with providers and discuss practical challenges. We invited key partners who provided other services to monthly MOMI PODS team meetings to share how we can better coordinate to meet patients' holistic needs. We also held monthly or weekly meetings among key MOMI PODS teams, including clinical; research; evaluation; data; budgeting; and development, recruitment, and design. While agendas varied across meetings, common points of discussion included: (a) identifying and addressing patient needs, (b) coordinating best care practices, (c) informing design from findings from the literature review, (d) targeting outcomes of interest and coordinating data sources for outcome measurement, (e) marketing and outreach efforts, (f) training needs of providers and patients, and (g) planning for evaluation of program success and dissemination of results.

### 2.2 Phase 2: identify MOMI PODS best practices of coordinating care for high-risk mothers

Our MOMI PODS stakeholder group collectively held over 50 years of experience in perinatal clinical care delivery and over 30 years of experience in maternal healthcare research. Over the course of 24 months, our MOMI PODS stakeholders met and leveraged their extensive expertise to review clinical activities provided within our GDM dyadic postpartum primary care pilot and other postpartum care programs within the institution and the state. Our team members not only attended internal group meetings, but also attended key institutional and state stakeholder meetings to share their experiences, learn from others' experiences, and generate a list of best care coordination practices that were being implemented by other postpartum care programs to connect high-risk mothers.

The stakeholders informed the implementation process (7) by indicating that a single medical home for the mother-baby dyad was a key component of practice that needed to be integrated into MOMI PODS. Connecting the mother-baby dyad to one medical home would increase high-risk mothers' access to primary care services for themselves and their babies simultaneously, including for postpartum screening for mood and anxiety disorders, blood pressure monitoring, glucose monitoring, and other preventative care. Another best practice of MOMI PODS was establishing care *before* delivery to help streamline services after birth when possible, to allow for establishment of clinical relationship prior to delivery. This care model was to be applied for the first 1,000 days of the baby's life given that this is a critical postpartum time period for the health and wellness status (8) of the mother-baby dyad. After this period, while dyads were transitioned out of the MOMI PODS program, they could continue to follow with the same PCP in the typical clinic structure.

### 2.3. Phase 3: design a MOMI PODS evaluation strategy

In concert with Phase 2 activities, our stakeholders conducted a scoping review to identify primary care interventions that have been implemented following high-risk pregnancy. Informed by the Chronic Care Model (9), our team's completed search strategy and synthesized the available literature with input from perinatal care providers and patients; our stakeholder team then collaborated to achieve consensus on the postpartum outcomes that would serve as the primary targets of the MOMI PODS program and determined the data sources needed to monitor outcomes consistently. This phase also informed ongoing quality improvement efforts and helped to establish program goals regarding how best-practice care would be defined and reported.

Our literature review identified that infant well-being is directly tied to parental well-being, which underscores that postpartum follow-up care is as essential for infants as it is for their birthing parent (10–13). This finding was consistent with the experiences of our stakeholder group. Our literature review also identified the individuals who are likely high-risk for poorer health outcomes and should be appropriate for the MOMI PODS programs. As such, the MOMI PODS target population was identified as pregnant or postpartum individuals who had experienced a complication of pregnancy (i.e., preterm birth, a hypertensive disorder of pregnancy, gestational diabetes mellitus), or who had a major chronic condition that had complicated the pregnancy (i.e., chronic hypertension, Type I or Type II diabetes mellitus), and those with chronic depression/anxiety or postpartum depression. Lastly, considering the evidence on maternal and infant health in the first 1,000 days of life (14), target outcomes of the MOMI PODS program were to: (a) decrease mother and infant morbidity, (b) proactively improve health outcomes for mom *and* baby via preventative care and chronic disease management, and (c) improve overall well-being. It was determined to evaluate this by Emergency Department utilization/recurrent hospitalization, adherence to preventative care and screening guidelines, and qualitative analysis of participants' experience of the program.

### 2.4. Phase 4: design MOMI PODS and MOMI PODS promotional materials

MOMI PODS was designed based on best care coordination practices identified by our stakeholders for the mother-baby dyad in the first 1,000 days of life. Given that MOMI PODS was a new, innovative program being launched in one academic medical center, marketing materials needed to be developed for pregnant and postpartum individuals as well as for clinicians who could initiate MOMI PODS referrals.

**Figure 2:**
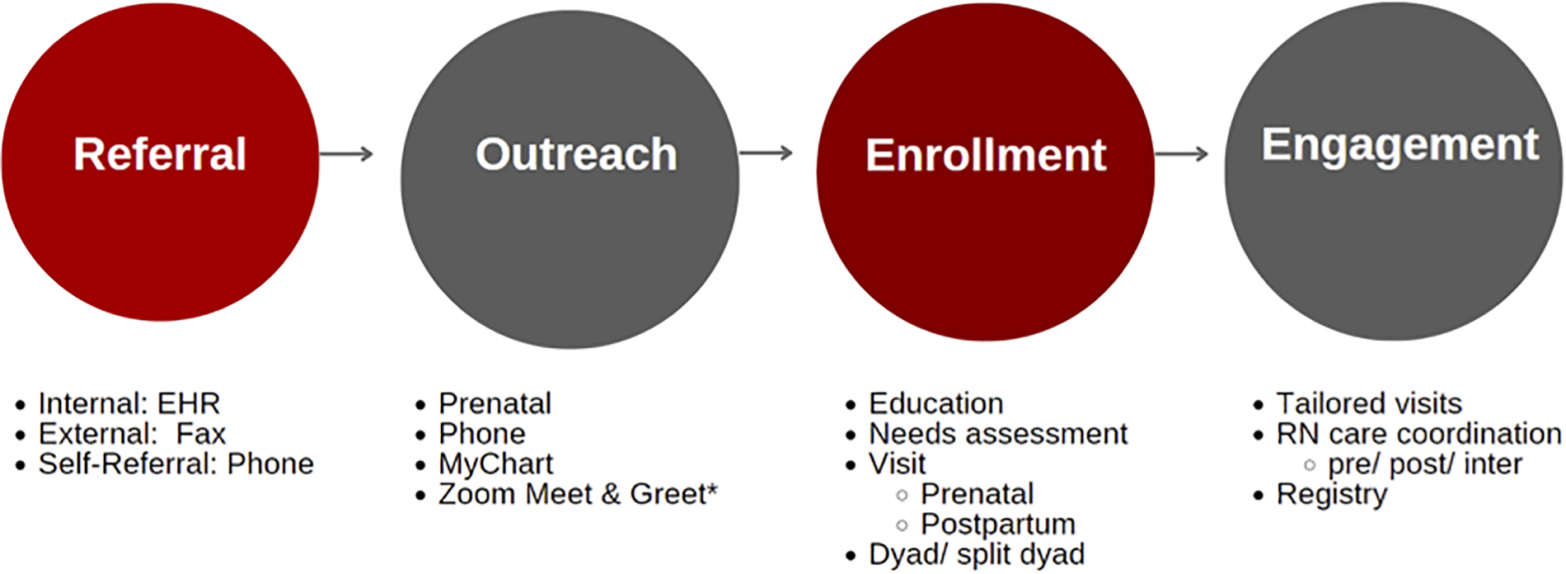
Clinical process overview for MOMI PODS.

The process through which mother-baby dyads enrolled in MOMI-PODS is described in Figure 2. Referrals were received from obstetric providers, social workers, or community resources (e.g., Women, Infant, Children supplemental nutrition program); the nurse coordinator reached out to referred patients on at least 3 separate encounters. Repeated attempts to enroll patients was a key part of the recruitment design, as barriers to phone calls throughout the workday, changes in schedules, as well as frequent appointments during pregnancy were all identified as potential barriers to enrollment. Once a patient was reached by the nurse coordinator, they were informed about the program and asked if they were interested in participating. If so, the nurse care coordinator performed a needs assessment to determine if any community referrals were beneficial, provided individualized health education, and made an appointment for the patient with a MOMI PODS provider at the patient's preferred location.

Based on current evidence and input from the MOMI PODS stakeholders, the following are examples of services that were provided to the mother-baby dyad once enrolled in MOMI PODS: immunizations, health care screenings (e.g., for developmental delays for infants; type 2 diabetes screening for mothers), chronic disease management and prevention, education and counseling (e.g., smoking cessation, referrals to other perinatal support programs), and lactation support.

To promote the MOMI PODS program regionally, our team's marketing materials were shared with clinicians (both attendings and trainees) in the Departments of Family Medicine and Community Medicine and Obstetrics and Gynecology to support recruitment to the program. Our team also identified three external groups for patient recruitment: Women, Infants, and Children (WIC), Moms2B (community-based program for low-income women with prenatal educational sessions and resources), and Perinatal Outreach and Encouragement for Moms (POEM, a group offering peer support, mentoring, and support groups for parents with mental health concerns). We chose these groups because of their frequent contact with and familiarity with the MOMI PODS target population, identified above in Phase 3, and their understanding of the outer setting as identified by CFIR (7). They provided additional and supplemental holistic services to mothers and babies including peer support, financial support for nutrition and basic needs, and mental health support. Marketing materials included a provider “quick reference guide,” a provider flyer describing “why” and how the MOMI PODS program works, a promotional flyer for potential participants to learn about the program, and a welcome letter for those who were referred (Supplementary Figure 1).

### 2.5. Phase 5: train MOMI PODS partners and providers

To enroll in the MOMI PODS program, individuals who were pregnant or had just given birth were referred to MOMI PODS by a care provider. Accordingly, our team developed training for clinicians and partners who work with our target population that explained MOMI PODS, described mother-baby dyads who would be eligible and appropriate for MOMI PODS care, and demonstrated how to make a MOMI PODS referral. Additionally, MOMI PODS providers were provided with materials reviewing best practices for follow up of pregnancy complications and postpartum care.

Additionally, innovative processes had to be developed to recruit and onboard MOMI PODS providers and unify intervention delivery. To enroll a practice as a MOMI PODS location, the MOMI PODS nurse coordinator met with the lead provider and practice manager at an interested clinic to explain the mission and vision of MOMI PODS. If the clinic expressed interest in moving forward, the nurse coordinator then connected with providers at that clinic to go over the same information to see if the individual providers wanted to provide care for MOMI PODS patients. Any provider that agreed to do so was then scheduled for one-on-one onboarding (i.e., peripartum best practices, education materials, referrals, roles of MOMI PODS team members, differences in scheduling). The nurse coordinator also met with 2–3 staff members for enrolled clinics so they could be trained in the specific scheduling needs and processes for this population. Our team met with 21 staff and 49 providers (physicians and advanced practice providers) to explain the purpose of MOMI PODS, including the benefits of making primary care services more accessible to the mother-baby dyad. This training was supplemented with provider “need-to-know” sheets that reviewed the description of MOMI PODS. For providers within Ohio State, we also assisted with creating “SmartPhrases” in the Epic-based electronic medical record to ensure uniformity and prompted providers to consider making referrals to essential community resources associated with MOMI PODS (Supplementary Figure 2). This served as a checklist for providers to help ensure uniformity of care and follow-up on any gaps or referrals. To ensure utilization, nurse coordinators sent providers a staff message the day prior to a dyad appointment to remind them they are seeing a patient enrolled in MOMI PODS and how to use the SmartPhrase. In addition, providers were trained about the essential gaps addressed by MOMI PODS to help create buy-in and emphasize the importance of each element of care.

### 2.6. Phase 6: pilot test MOMI PODS

MOMI-PODS officially launched in December 2021 with the goal of enrolling 1,260 mother-baby dyads by 2024. In addition to monitoring our enrollment goals, we have also documented barriers to MOMI PODS implementation and opportunities for program improvement. From December 2021 to December 2022, a total of 216 mother-baby dyads have been referred to MOMI PODS. Of these, 123 dyads enrolled, 15 declined, and 78 did not respond to outreach calls from the clinical nurse care coordinator. Of those who declined, 12 stated that they and/or their baby already had a primary care doctor. Data collection and evaluation efforts are currently underway to monitor the impact of MOMI PODS on mother-baby morbidity, well-being, and overall health outcomes. Patient satisfaction surveys were completed in October 2022 and are currently being analyzed. Two patients enrolled in MOMI PODS were also recruited to join the advisory council to inform further development. Qualitative analysis of the patient experience with MOMI PODS is currently underway.

## 3. Discussion

The first 1,000 days of life is a critical period for the health and wellness of the mother-baby dyad, particularly for high-risk mothers who do not have established primary care providers or are affected by chronic health conditions. MOMI-PODS offers an innovative care model to ensure that the perinatal health needs of the mother-baby dyad are appropriately evaluated and met. Since its launch, the MOMI-PODS program has been disseminated to maternal care groups in the central Ohio region with the goal to expand MOMI-PODS' reach. Successful expansion of MOMI-PODS, however, will be dependent on our team's ability to tackle several notable barriers to MOMI-PODS implementation. Below, we describe these barriers and the strategies we have deployed to overcome these care challenges.

### 3.1. Barriers to care and strategies to overcome

Through our experiences piloting the MOMI PODS program, our team encountered several barriers at the provider-, clinic-, and community-levels that have impeded program implementation. These barriers related to: (a) provider capacity, (b) response to referral, (c) consistent provision of care, (d) scheduling of dyad visits, (e) data tracking and integration, and (f) circumstances requiring special accommodations. In light of our identified barriers, we have planned and/or deployed several strategies to support successful implementation of MOMI PODS going forward.

### 3.2. Provider capacity

When MOMI PODS was first initiated in December 2021, three individual clinicians were appointed to serve as MOMI PODS providers and were designated to travel to MOMI PODS sites for patient encounters. However, staffing shortages associated with the COVID-19 pandemic substantially reduced the proportion of clinicians who could dedicate adequate time to MOMI PODS encounters, especially when these encounters required regional travel. Furthermore, our larger health system was committed to building capacity of their clinicians to provide evidence-based, patient-centered care in general, but this sometimes led to clinicians participating in general “capacity building” activities and events, with the unintended consequence of reducing the amount of time allocated to capacity building in delivering MOMI PODS in particular.

**Figure 3:**
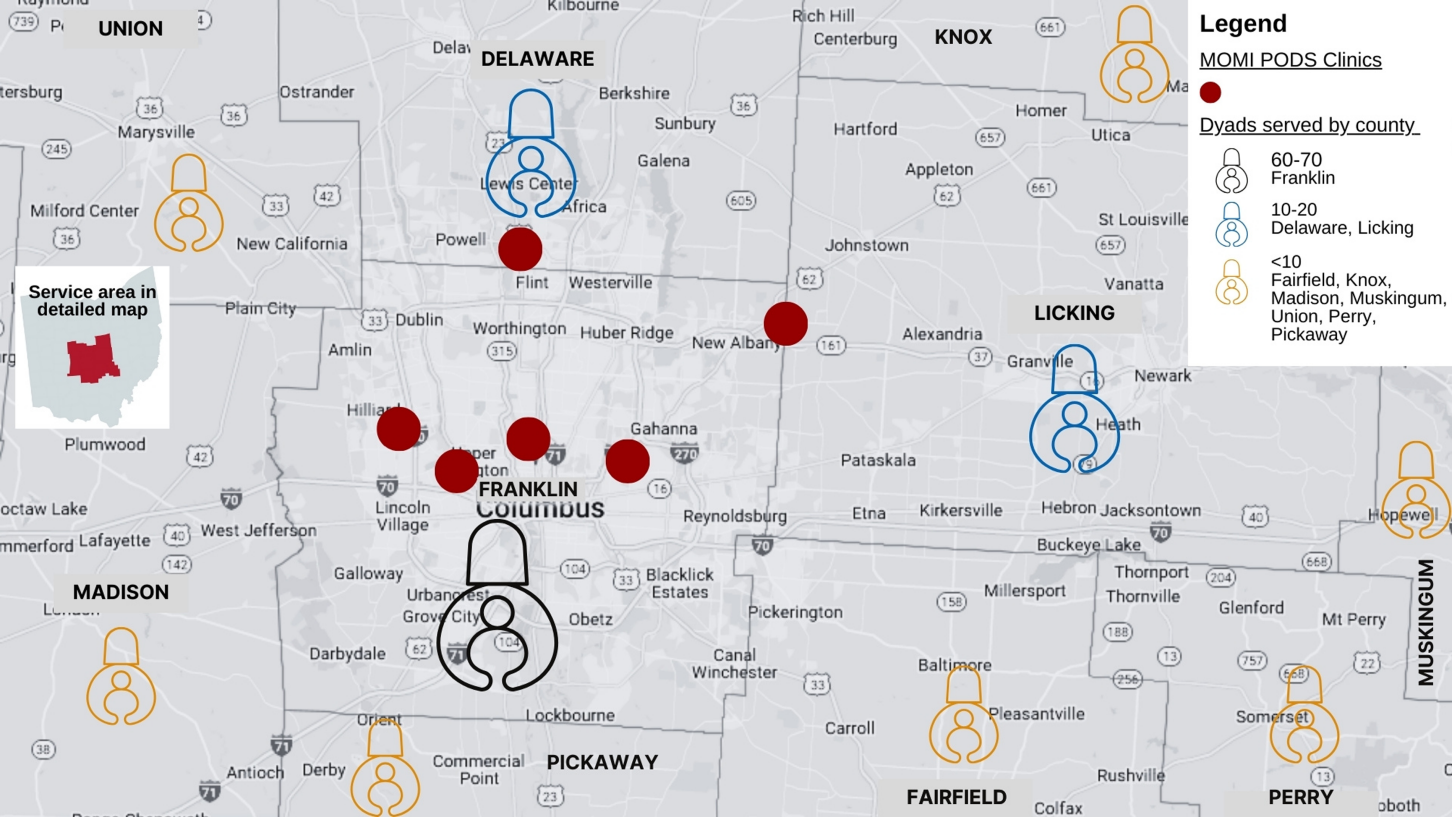
MOMI PODS clinic and patient service area geography.

To build provider capacity for providing services to the mother-baby dyad, we held eight Meet & Greet sessions with seven primary care clinics who all enrolled as MOMI PODS sites. These clinics represent six diverse neighborhoods within Franklin County (Figure 3). The purpose of these sessions was to share the structure and purpose of MOMI PODS, present preliminary outcomes from the GDM pilot (4) and identify additional providers who would be willing to partner with our team with the goal of expanding the reach of MOMI PODS services to other regions in Ohio.

### 3.3. Response to referral

In total, 36% of patients with placed referrals never responded to contact attempts. Some portion of this loss was due to incomplete or inaccurate contact information on referral forms. Some patients also initially accepted the referral to establish care post-delivery, however would then not respond to attempts to schedule or establish care postpartum.

A standardized nursing workflow of three nursing outreach attempts for all patients was established to offer multiple opportunities to enroll for interested patients. Referrals could be placed at any time during pregnancy; patients were encouraged to establish care pre-delivery whenever possible to foster an established relationship and encourage postpartum follow up. For patients who accepted referral but planned to schedule their first visit with their newborn, nursing outreach was planned 30 days and 14 days before the patient's due date, as well as after delivery but before discharge home. In the case of early delivery or postpartum referral placement, nursing outreach occurred as soon as possible after delivery.

### 3.4. Consistent provision of care

Ideally, mother-baby dyads who enter the MOMI-PODS program should receive “high-touch” care, meaning that dyads would be seen frequently by providers to optimize both maternal and infant health outcomes. However, our patients frequently faced obstacles accessing services as regularly as intended. For instance, given that multiple conditions were addressed through MOMI PODS, dyads often needed to schedule appointments with multiple specialty providers (behavioral counseling, endocrinology, etc.). Scheduling and re-scheduling these appointments proved quite challenging given the difficulties of contacting different providers' offices, as not all providers were located at the same clinic site. Moreover, some patients relied on transportation assistance that was covered through Medicaid and other payers; coordinating transportation further complicated patients' scheduled visits and limited the extent to which patients were able to participate in MOMI PODS.

Our MOMI PODS team was not able to provide consistent continuity of care given challenges with patient scheduling and the complex needs of dyads that should be addressed by one or more specialty providers stemming from multiple clinic locations. To improve continuity of care and maintain the “high touch” focus of the MOMI PODS program, we appointed one dedicated nursing staff member to manage MOMI PODS call lines. The goal of these designated MOMI PODS call lines was to minimize burden on patients and reduce the number of phone calls needed to schedule specialty appointments. Other strategies to support continuity of care included sending text, phone, and email reminders to patients to attend their appointments, evaluating the social needs of patients during their intake assessment to understand barriers to care, and establishing partnerships with rideshare services, particularly for patients whose appointments could not be accommodated by Medicaid-supported transportation due to a short turnaround time. Nursing staff helped to individualize patient support and acted as liaisons between the patient with the larger clinical team. A co-disciplinary effort to partner with a mobile unit to provide dyadic interventions, such as breastfeeding support, was also initiated.

### 3.5. Scheduling of dyad visits

Relatedly, dyadic visits (back-to-back appointments for parent and infant) were difficult to schedule within the standard clinic workflow. Dyad appointments were typically scheduled for longer durations of time to adequately assess and manage the needs of both mother and baby. Without blocks of time reserved for these longer patient encounters, fewer dyads could be scheduled around individual (i.e., non-dyad) patient appointments—or the standard clinic workflow.

By establishing a designated MOMI PODS call line, our patients were able to immediately speak with a MOMI PODS clinical nurse care coordinator, thereby streamlining the appointment scheduling process. Further, we worked with our health system to build “blocked” dyad timeslots directly within the scheduling program. This allowed clinicians and staff to easily identify where dyads could be inserted into the schedule and preserved longer appointment blocks that could not be scheduled by non-dyad patients. Each clinician and office were able to tailor their schedules to indicate how many dyads could be seen each week and the duration of each encounter.

### 3.6. Data tracking and integration

Of paramount importance to the MOMI-PODS program is the health outcomes of both the mother and infant. Despite the value of the combined dyad encounter, our team identified several obstacles to track patient data and monitor patient outcomes. These obstacles were primarily attributed to the fragmentation of how data were entered into patient charts. For instance, many points of data—such as delivery complications—were spread across mother and infant charts. One main data point that our team attempted to track was the count of engagements our clinical team had with postpartum individuals, however this tracked encounters in infants' charts only. This limitation in our data tracking methods reduced our ability to accurately calculate the number of clinician encounters patients received. Further, EHR data mining limitations prevented our centralized data team from accessing patient data, inhibiting their ability to link patient data to Medicaid claims data. This prevented us from analyzing Medicaid billing data and determining the healthcare utilization trends (e.g., hospitalization, emergency department use) of our patients.

To optimize data tracking and integration, we created a dashboard for the clinical nurse care coordinator to monitor patient check-ins and follow-up appointments. We also held bimonthly meetings with our data team to identify solutions to data tracking and integration barriers. We explored options for co-appointing members of our health system's data team to be embedded members of the MOMI PODS program such that they would develop a deeper understanding of our data tracking processes and potential options for improvement.

### 3.7. Unexpected circumstances

We also encountered several unique scenarios that required special accommodations. Examples of these circumstances included evaluating and treating a set of quadruplets with highly variable needs (while also addressing the mother's health needs) and attempting to deliver services to patients residing in rural areas with intermittent access to reliable, local maternal care, as our MOMI PODS program was restricted to the metropolitan area of Columbus, Ohio.

As part of our dyad scheduling “blocks,” we also built templates that allowed for staff to schedule appointments for parent-infant dyads who required more time during their appointments. This allowed our team to have sufficient time dedicated to treat the needs of patients with circumstances that often extended the length of their appointments. To further enhance care for non-English speaking patients, we determined their language preferences immediately during their intake assessment, which enabled our team to plan for future visits and ensure that proper interpreter services were coordinated. For our rural patients, we have initiated efforts to expand our services to our affiliated federally qualified healthcare centers (FQHCs), which has allowed us to have a broader reach. We also added a telehealth option for patients with broadband access, providing more flexibility in care access options.

### 3.8. Case example of successful delivery

A 28-year-old female with GDM was referred to MOMI PODS at 28 weeks' gestation for follow up for her and her infant. While she reported feeling well when she was present at her infant's newborn visit, when she was seen in a dyad visit at 4 weeks postpartum she reported significant anxiety, with an Edinburgh Postnatal Depression Scale (EPDS) score of 8. She was referred to postpartum counseling resources and connected with cognitive behavioral therapy, which initially improved her anxiety. However, she was found to have worsened anxiety when seen for her infant's four month well child visit, notably a time she would not have had scheduled follow up otherwise. At this time, she was initiated on selective serotonin reuptake inhibitor (SSRI) therapy, with significant improvement in her anxiety noted at follow up. At her last primary care visit at 1 year postpartum, her Generalized Anxiety Disorder (GAD)-7 scale score was 2, indicating minimal anxiety. During her postpartum course, she also received MOMI PODS lactation support, completed all recommended postpartum screenings based on her clinical profile (e.g., glucose screening following GDM), and was connected with several community resources. She had a total of six primary care visits in her first year postpartum and 35 nurse and social work patient outreach encounters, and continues to be actively engaged in her own care as well as that of her infant. MOMI PODS not only facilitated this patient's access to treatment, but also facilitated guideline-concordant care.

## 4. Conclusion

Reductions in maternal and infant morbidity and mortality will require changes to outpatient structures to promote mother and infant wellbeing and rapidly address threats to postpartum health. We have implemented an innovative mother-infant dyad program to connect high-risk mothers with primary care and community resources after pregnancy. This was achieved with the help of multiple stakeholders and a dedicated clinical team, who identified best practices, trained partners and providers, and continuously sought opportunities for improvement. Several barriers to MOMI PODS implementation were identified, including the overwhelming impact of the global COVID-19 pandemic. Such barriers were mitigated by focusing on provider capacity, employing inventive data tracking methods, and creating more flexible clinic templates. Through this unique interdisciplinary program, we hope to improve peripartum primary care access and promote maternal and child health. Critical next steps in this important line of work include continued evaluation of program feasibility, acceptability, and effectiveness and identification of the mechanisms by which MOMI PODS affects dyadic postpartum health, allowing us to optimize this innovative model of care.

## Data Availability

The institutional deidentified data underlying this article are available from the corresponding author on reasonable request and institutional approval. Interested parties will be required to complete an institutional Data Use Agreement, and data will be made available via Secure Data transfer after institutional approval is secured.

## References

[1] Masho SW Cha S Karjane N McGee E Charles R Hines L Correlates of postpartum visits among medicaid recipients: an analysis using claims data from a managed care organization. J Womens Health (Larchmt). (2018) 27(6):836–43. 10.1089/jwh.2016.6137 29451839

[2] Essien UR Molina RL Lasser KE. Strengthening the postpartum transition of care to address racial disparities in maternal health. J Natl Med Assoc. (2019) 111(4):349–51. 10.1016/j.jnma.2018.10.016 30503575

[3] Bennett WL Chang HY Levine DM Wang L Neale D Werner EF Utilization of primary and obstetric care after medically complicated pregnancies: an analysis of medical claims data. J Gen Intern Med. (2014) 29(4):636–45. 10.1007/s11606-013-2744-2 24474651 PMC3965743

[4] D’Amico R Dalmacy D Akinduro JA Hyer M Thung S Mao S Patterns of postpartum primary care follow-up and diabetes-related care after diagnosis of gestational diabetes. JAMA Netw Open. (2023) 6(2):e2254765. 10.1001/jamanetworkopen.2022.54765 36745454 PMC12512597

[5] Bose Brill S May S Lorenz AM Spence D Prater L Shellhaas C Mother-infant dyad program in primary care: evidence-based postpartum care following gestational diabetes. J Matern Fetal Neonatal Med. (2022) 35(25):9336–41. 10.1080/14767058.2022.2032633 35098857

[6] Brown JA Leonard M Clinton T Bower J Gillespie S Fareed N Mothers’ perspectives on a mother/infant dyad postpartum primary care program following gestational diabetes mellitus: a qualitative pilot study. Sci Diabetes Self Manag Care. (2022) 48(4):247–57. 10.1177/26350106221100539 35658777

[7] Damschroder LJ Aron DC Keith RE Kirsh SR Alexander JA Lowery JC. Fostering implementation of health services research findings into practice: a consolidated framework for advancing implementation science. Implement Sci. (2009) 4:50. 10.1186/1748-5908-4-50 19664226 PMC2736161

[8] Likhar A Patil MS. Importance of maternal nutrition in the first 1,000 days of life and its effects on child development: a narrative review. Cureus. (2022) 14(10):e30083. 10.7759/cureus.30083 36381799 PMC9640361

[9] Bose-Brill S Freeman TE Miles L Prater L Chang MW Bower JK. Bridging the gap in gestational diabetes: an interdisciplinary approach to improving GDM using a chronic care-based clinical framework. Am J Perinatol. (2020) 37(9):970–4. 10.1055/s-0039-1689002 31146292

[10] Perez-Lopez MJ Leyva-Resendiz IG Vazquez-Vega B Chavez-Lopez EL Hernandez-Rivera JC Paniagua-Sierra JR. Perinatal morbidity and mortality of children born to mothers with chronic kidney disease. Gac Med Mex. (2021) 157(4):356–63; Morbilidad y mortalidad perinatal de hijos de mujeres con enfermedad renal cronica. 10.24875/GMM.M21000575 35133329

[11] Guo VYW Wong CKH Wong RSM Yu EYT Ip P Lam CLK. Spillover effects of maternal chronic disease on children’s quality of life and behaviors among low-income families. Patient. (2018) 11(6):625–35. 10.1007/s40271-018-0314-8 29777517

[12] Guyon-Harris KL Bogen DL Huth-Bocks AC. Maternal psychological well-being and infant emergency department utilization. Acad Pediatr. (2021) 21(5):885–91. 10.1016/j.acap.2021.01.021 33548524

[13] Barnes J Theule J. Maternal depression and infant attachment security: a meta-analysis. Infant Ment Health J. (2019) 40(6):817–34. 10.1002/imhj.21812 31415711

[14] Why 1,000 Days. *FHI solutions.* Available at: https://thousanddays.org/why-1000-days/ (Accessed May 30, 2023).

