# Supplementary material for: Developing a multimodal maternal infant perinatal outpatient delivery system: the MOMI PODS program
Source: Front Glob Womens Health. 2023 Sep 21;4:1232662. doi: 10.3389/fgwh.2023.1232662 (PMC10552521; doi:10.3389/fgwh.2023.1232662)
Supplement: Supplementary file 1 [file Datasheet1.pdf]

Supplementary Material

Developing a Multimodal Maternal Infant Perinatal Outpatient Delivery System: the MOMI PODS Program

Seuli Bose Brill, Lisa A Jucket, Rachel D’Amico*, et al.

*** Correspondence:** Rachel D’Amico: Rachel.d’amico@osumc.edu

1.1 Supplementary Figures

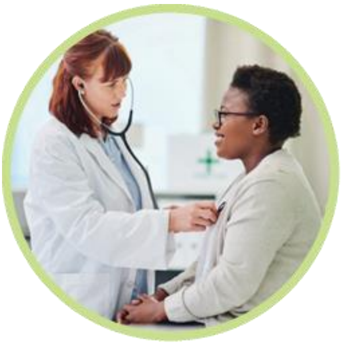

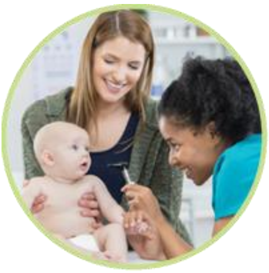

momi pods

KEEPING MOM + BABY TOGETHER IN PRIMARY CARE

Congratulations on the upcoming birth of your new baby!
You have been referred to our MOMI PODS program. Our physicians can provide primary care for both you and your new baby.

You and your baby can have the same primary care team, including the option of combined visits!

You should continue to see your OB/GYN for your post-partum visits, but it is important for you to have a primary care doctor who will take care of you after you have completed all necessary obstetric care.

A healthy baby requires a healthy mom.

Many women put their health on the back burner after the birth of their baby, but it is important for women to maintain regular appointments. Our goal is to make access to primary care more convenient.
A primary care doctor (PCP) helps you stay healthy (prevent illness) and helps take care of any health problems you are having.

- Women need special support as they recover from pregnancy. Our PCPs are experts in providing primary care for women after pregnancy. They can take care of the unique postpartum primary care needs that arise.
- Our PCPs can provide pediatric care for your baby, such as well child checks to ensure good growth and development, sick visits, and immunizations.

By taking care of both you and your baby, your PCP has unique understanding of the challenges you face as a new mom. Having the same PCP for you and your baby also makes it more convenient for you to access care, such as having your appointments scheduled at the same time as your baby.
If you are interested in being seen at one of our clinics, please call us at **614-XXX-XXXX** and mention you have been referred to the MOMI PODS program. You will speak with our Clinical Nurse Care Coordinator who will:

- Explain the benefits of joining the program.
- Help you schedule your first clinic visit.
- Answer any questions you may have.

We look forward to meeting you!

THE OHIO STATE UNIVERSITY
WEXNER MEDICAL CENTER

MOMI PODS: Keeping Mom + Baby **Together** in **Primary Care**

Supplementary Figure 1. Welcome letter for recruited patients to the MOMI PODS program.

DYAD Maternal Screening

Was the pregnancy affected by Diabetes? Yes,

- Resources provided: Booklet: 'Gestational Diabetes Mellitus Toolkit'
  - Was this Gestational diabetes? Yes, T2DM screening: 2 hour OGTT ordered & scheduling information given (4-12 weeks postpartum).

Postpartum Physical Activity

- Engagement in physical activity: No, does not engage in regular physical activity.
- Provided guidance about benefits of exercise: Encouraged mother to engage in physical activity per the CDC guidelines for adults (ages 18-64 years): At least 150 minutes a week of moderate intensity activity such as brisk walking. At least 2 days a week of activities that strengthen muscles.

Postpartum Care & Family Planning

- Did patient attend OB post-partum visit? yes
- Education provided on safe spacing of pregnancy 18mo from birth to conception? yes
- Current method of family planning: OCPs or POPs
- Education provided on following methods: LARC (IUD or Nexplanon)

Breastfeeding / Nutrition

  - What does child eat? Breast milk. Any difficulties with breastfeeding? Yes, Has patient been referred for a lactation consult? Ordered referral for OSU lactation ambulatory consult (AMB REFERRAL TO LACTATION).
  - Education handouts provided? WIC Breastfeeding Support Handout.
- Is a consult with a registered dietitian indicated? no

Mental Health

- Patient received mental health screening Yes, What tools did you use? EPDS completed
  - Was the screening positive? Yes (EPDS>=10, PHQ9>10, GAD7>10),
    - Was she given/referred to community resource(s)? Postpartum Depression Action Sheet by March of Dimes provided., POEM handout (Perinatal Outreach & Encouragement for Moms) provided. and Maternal Mental Health Hotline Handout (HRSA) provided.
    - Was mental health treated at today's visit? yes

Smoking

- Current Status: Current smoker.

Explained benefits of smoking cessation & provided resources: Handout: Secondhand Tobacco Smoke and the Health of Your Family by the EPA.

Supplemental Figure 2: Provider example SmartPhrase for maternal care

MOMI PODS SMART PHRASE:

- There is 1 smart phrase to be used for maternal screening at each WCC if mom brings child to the appointment:

.DYADMATERNALSCREENING

Name

DYADMATERNALSCREEN

Description

MOMI PODS
- If mom is being seen for an Office Visit, include in mom's
- At WCC, if mom is present and NOT being seen, include
Visit Note
-Split Dyads: Use at each office visit

- We start using the smart phrase at the 1 mo WCC. It covers topics that affect mom *and* baby.
  - At WCC – if mom is being seen for an Office Visit, include the phrase in your documentation for mom's Office Visit Note
  - At WCC – if mom is NOT being seen, include the smart phrase in your documentation for child's Office Visit Note
- This same smart phrase will be used at each split dyad visit as well.

DYAD Maternal Screening

Was the pregnancy affected by Diabetes? {yes/no diabetes:42047}

Postpartum Physical Activity
- Engagement in physical activity: {physical activity:39257}
- Provided guidance about benefits of exercise: {exercise benefits:39563}

Postpartum Care & Family Planning
- Did patient attend OB post-partum visit? {YES/NO:22071}
- Education provided on safe spacing of pregnancy 18mo from birth to conception? {YES/NO:22071}
- Current method of family planning: {Family Planning Options:42031}
- Education provided on following methods: {Family Planning Education:42032}

Breastfeeding / Nutrition
- What does child eat? {feeding options:40656}
- Is a consult with a registered dietitian indicated? {YES/NO:22071}

Mental Health
- Patient received mental health screening {Screened mental health?:42050}

Smoking
- Current Status: {smoking status/history:40820}
